# LDLRAD4 is a potential diagnostic and prognostic biomarker correlated with immune infiltration in myelodysplastic syndromes

**DOI:** 10.3389/fgene.2025.1540161

**Published:** 2025-10-29

**Authors:** Mengjie Xu, Shihao Wu, Kaixiang Zhang, Lirong Nie, Qinghua Li, Jihong Zhong, Yuming Zhang, Honghua He

**Affiliations:** ^1^ Department of Hematology, Affiliated Hospital of Guangdong Medical University, Zhanjiang, China; ^2^ Guangdong Medical University, Zhanjiang, China

**Keywords:** myelodysplastic syndromes, biological markers, machine learning, LDLRAD4, immune infiltration

## Abstract

**Purpose:**

Myelodysplastic syndromes (MDS) are a group of hematological disorders that remain relatively under-explored, which are characterized by inconspicuous early symptoms and generally poor prognosis. Owing to the complex and variable pathogenesis of MDS, there is a relative paucity of available therapeutic options. Consequently, in-depth investigation into the pathogenesis of MDS and the search for effective targeted therapies have become urgent priorities.

**Methods:**

In this study, we leveraged the Gene Expression Omnibus (GEO) database to identify differentially expressed genes (DEGs) and conducted functional enrichment analysis. Utilizing three machine learning algorithms—Least Absolute Shrinkage and Selection Operator (LASSO), Support Vector Machine Recursive Feature Elimination (SVM-RFE), and Random Forest (RF)—we pinpointed hub genes. Furthermore, this study explored the relationship between hub gene expression levels and immune infiltration.

**Results:**

Our analysis identified three hub genes: LDLRAD4, FAM43A, and KCNK5, with LDLRAD4 showing a close association with TGF-β and MAPK signaling pathways. Furthermore, this study revealed a positive correlation between LDLRAD4 expression levels and immune infiltration, particularly with natural killer (NK) cells, offering a novel immunological perspective on LDLRAD4. Ultimately, we observed that the overexpression of LDLRAD4 can suppress the proliferative capacity of MDS cells, induce cell cycle arrest, and enhance apoptosis.

**Conclusion:**

We conclude that LDLRAD4, FAM43A, and KCNK5 are potential biomarkers for MDS. LDLRAD4’s overexpression *in vitro* inhibits MDS cell proliferation and promotes apoptosis, suggesting significant potential for immunotherapy research. These findings collectively identify LDLRAD4 as a promising therapeutic target for MDS. However, its clinical applicability warrants further investigation to validate its potential.

## Introduction

Myelodysplastic syndromes (MDS) comprise a group of malignant clonal diseases originating from hematopoietic stem cells (HSCs). They are characterized by ineffective hematopoiesis, abnormal bone marrow proliferation, a reduction in peripheral blood cell numbers, and an increased risk of transformation into acute myeloid leukemia (AML) (Bazinet and Bravo, 2022),Currently, therapeutic strategies for MDS are evolving with increasing differentiation. For patients in the lower-risk group, there is an adoption of new strategies targeting inflammatory immune pathways. For patients in the higher-risk group, various approaches are employed merely to delay progression. Allogeneic hematopoietic stem cell transplantation represents the only curative method for MDS, yet its applicability is limited by factors such as the patient’s physical condition, familial economic status, and donor availability, precluding its universal application.

The pathogenesis of this disease is multifaceted, involving a myriad of factors. In function in MDS patients. Scholars believe that the pathogenesis of this disease is intricately linked not only to cell biology, epigenetics, and similar fields but also to abnormal immune system function recent years, increased scholarly focus has been placed on the abnormal immune (Trowbridge and Starczynowski, 2021; Wang et al., 2018; Kapoor et al., 2021). This study aims to conduct a comprehensive investigation into the pathogenesis of the disease from an immunological perspective and to develop more effective immunotherapies. Furthermore, mounting evidence suggests that MDS is linked to abnormal gene mutations (Ogawa, 2019),Therefore, identifying additional molecular biomarkers and exploring their potential applications in immunotherapy are crucial for the early diagnosis, treatment, and prognosis assessment of MDS. These studies aim to enhance understanding of the pathogenesis of MDS and to provide more precise treatment solutions. This approach is anticipated to help slow down disease progression, maintain and improve patients’ quality of life, and increase their survival rate.

The use of machine learning (ML) in biomedical fields has escalated with the rapid development of gene microarray and high-throughput technologies. ML’s powerful data processing and pattern recognition capabilities have facilitated significant progress in analyzing large data sets and discovering valuable relationships (Greener et al., 2022; Peiffer-Smadja et al., 2020; Eraslan et al., 2019). This study integrates bioinformatics and machine learning to enhance the accuracy and predictability of MDS diagnosis. Gene expression matrices of MDS patients were obtained from the GEO database, and differential expression and enrichment analyses were performed.; Subsequently, LASSO regression, Random Forest, and SVM-REF were utilized to screen for pivotal genes. MDS patients were then grouped based on the expression of these pivotal genes, and further differential expression and enrichment analyses were conducted to gain insight into the different gene functions and regulatory mechanisms at varying expression levels. Finally, the CIBERSORT algorithm, based on gene expression profiling, was utilized in this study to quantify the infiltration of immune cells, evaluate the correlation between immune cells in MDS and normal samples, and investigate the correlation between the immune functions of key genes and immune cells.

## Materials and methods

### Microarray chip data information

GEO (http://www.ncbi.nlm.nih.gov/geo), a public genomics data repository, is created and maintained by the National Center for Biotechnology Information (NCBI) and includes high-throughput gene expression data, chips, and microarrays. The gene expression profiles of MDS, namely, GSE4619, GSE19429, and GSE58831, were sourced and downloaded from GEO. The aforementioned validation set dataset comprises 397 MDS patients and 35 normal healthy controls. GSE2779, utilized as a validation set, includes 159 MDS patients and 17 normal healthy controls.

### Data preprocessing

Initially, the GEOquery package was used to convert the probe matrix to the gene matrix, incorporating the probe annotation file in the process. In instances where multiple probes corresponded to the same gene, the expression value of the gene was determined by calculating the average value of the probes. To mitigate the batch effects arising from different platforms, the sva package was employed, given that the datasets originated from various sources.

### Identification of differentially expressed genes

Analysis of DEGs between MDS patients and normal controls was conducted using the limma package. The selection criteria for DEGs included a p-value <0.05 and an absolute fold change (FC) > 1.

### Gene set enrichment analysis assessment

The enrichment of Gene Ontology (GO) and Kyoto Encyclopedia of Genes and Genomes (KEGG) pathways was analyzed using the R package ‘clusterProfiler’. Gene Set Enrichment Analysis (GSEA) and Gene Set Variation Analysis (GSVA) were utilized to investigate differences in biological functions among various expression groups of hub genes, thereby revealing their underlying mechanisms.

### Hub gene LASSO regression screening

LASSO regression, a machine learning algorithm, is commonly used for fitting generalized linear models. This algorithm is distinguished by its ability to perform variable selection and complexity regularization simultaneously (Cheung-Lee and Link, 2019). The parameter λ adjusts the complexity level, whereby higher values impose a greater penalty on linear models with many variables. This approach yields a smaller number of selected genes, producing a more concise and representative set of key genes. The glmnet package in R was utilized in this study for LASSO regression analysis of candidate hub genes. The optimal value of λ was determined via 10-fold cross-validation, by selecting the value that produced the smallest criterion.

### Hub gene random forest model screening

The random forest model, a machine learning technique, employs multiple independent decision trees for predicting classification or regression (Rigatti, 2017). In this study, the R package ‘randomforest’ was utilized to construct the random forest model. The optimal number of variables was determined by calculating the average error rate of candidate hub genes. Subsequently, the error rate was evaluated for tree numbers ranging from 1 to 500, selecting the number with the lowest error rate. Following the determination of the parameters, the random forest tree model was constructed. The feature importance score for each candidate hub gene was then determined, selecting genes with an importance value greater than 1.

### Hub gene SVM-RFE screening

SVM-RFE is a widely utilized supervised machine learning protocol for classification and regression (Frost and Amos, 2017). The “e1071” package in R was used, applying the support vector machine recursive feature elimination (SVM-RFE) algorithm based on nonlinear support vector machines to identify hub genes.

### Diagnostic value of hub genes in MDS

To assess the accuracy of the hub genes identified by machine learning, ROC curves were generated for MDS patients and normal controls in the training group. A larger area under the curve (AUC) indicates higher accuracy of the gene as a hub gene in MDS. This method’s effectiveness was further validated in the validation group.

### Identification of immune cell infiltration

The CIBERSORT algorithm was employed to calculate the differential abundance of 22 types of immune infiltrating cells. Heatmaps and violin plots displaying the correlation of immune cells were prepared using the R packages “corrplot” and “ggplot2”. This study utilized the CIBERSORT algorithm to determine the relative proportion of different immune cells in MDS and normal control groups.

### Correlation analysis between hub genes and infiltrating immune cells

Analysis of the correlation between hub genes and immune cells was conducted using the Spearman correlation coefficient.

### Cell culture

The human MDS cell lines, SKM-1, were acquired from Yuchi Biotechnology Ltd. (Shanghai, China) and cultured in Dulbecco’s modified Eagle medium supplemented with 10% fetal bovine serum. These cells were maintained at 37 °C in a cell culture incubator with 5% CO_2_.

### RNA extraction and quantitative real time polymerase chain reaction (qRT-PCR)

Total RNA was extracted using TRIzol reagent and processed for cDNA synthesize, adhering strictly to the manufacturer’s protocol (Cat: 11119ES60, YEASEN Biotechnology Ltd., Shanghai, China). The resulting cDNA was then subjected to RT-PCR using QuantStudio 6 Pro (Thermo Fisher Scientific, Waltham, MA, United States) in conjunction with SYBR Green PCR Master Mix. Fold changes in mean values were determined using the double delta CT method. Each experiment yielded three independent datasets. The primer sequences were as follows: *LDLRAD4*: 5′- GTT​GCA​CTT​AGG​CTG​GGT​CT -3′ (F); 5′- AGG​TGA​GGG​GCA​GAG​AGA​AA-3′ (R). *GADPH*:5′-GGAGCGAGATCCCTCCAAAAT-3′(F); 5′-GGC​TGT​TGT​CAT​ACT​TCT​CAT​GG-3′ (R).

### Western blotting

Cells were harvested and incubated with RIPA lysis buffer on ice for 30 min to extract total protein. Following centrifugation at 12,000 rpm for 15 min at a temperature of 4 °C, the protein-rich supernatant was gathered. The samples were then applied to a gel for sodium dodecyl-sulfate polyacrylamide gel electrophoresis and subsequently transferred onto a 0.22-μm polyvinylidene difluoride membrane. To block the membrane, 5% skimmed milk was used at a temperature range of 24 °C–30 °C for 30 min, followed by incubation with primary antibody at 4 °C. After three washes with PBS-Tween 20 (PBST), the membrane was incubated with a secondary antibody (Cat: SSA004, 1:3000, Sino Biological Inc., China) for 1 h at 24 °C–30 °C. After additional washes with PBST, the protein bands were detected using a Tanon-5200 image analyzer (Thermo Fisher Scientific, Waltham, MA, United States). ImageJ software was employed to analyze the film strips, normalizing the protein intensities to the corresponding β-actin bands. The antibodies used and their dilution ratios were as follows: anti-LDLRAD4 (Cat:PA5-70568, 1.0 μg/mL, Thermo Fisher Scientific, Waltham, MA, United States), anti-β-Tubulin (Cat: E021040-01, 1:5000, EarthOx, United States).

### Cell counting Kit-8 (CCK-8) assay

Transfected cells were distributed into 96-well plates at a density of 1,000 cells per well, containing 100 μL of medium in each. Following incubation periods of 24, 48, and 72 h, 10 μL of CCK-8 solution was introduced to each well, and the plates were incubated in the dark at 37 °C for 2 h. Subsequently, the absorbance at 450 nm was measured to assess cell viability.

### Cell cycle assay

Trypsin-treated cells were harvested, rinsed with PBS, suspended in pre-cooled 70% ethanol, and fixed at 4 °C for 2 h. Subsequently, the supernatant was aspirated, and the cells were washed again with PBS. Propidium iodide staining solution was then added, and the cells were incubated in the dark at 37 °C for 30 min.

### Flow cytometric analysis of cell apoptosis

Cells were digested and resuspended in 100 μL 1× binding buffer. Stained with 5 μL Annexin V and 10 μL PI (Cat:40302ES20,YEASEN, Shanghai, China) for 15 min at room temperature (37 °C) in the dark, and finally 400 μL 1× binding buffer was added. The number of apoptotic cells was analyzed by using FACS Aria flow cytometer with CellQuest software and the data were analyzed with FlowJo software.

### Cell transfection

SKM-1 were plated into 6-well plates and allowed to proliferate to a 50%–60% confluence prior to transfection. Cells were divided into negative control (pcDNA3.1-NC) and transfection (pcDNA3.1-LDLRAD4) groups. Transfection was performed following the instructions for Lipo8000 protocol (Beyotime Biotechnology Ltd., Shanghai, China). Eight hours later, the serum-free Opti-MEM was substituted with complete medium containing 10% serum. Cells were further incubated for 48 h at 37 °C with 5% CO2) after transfection.

### Statistical analysis

Statistical analysis of the data in this study was conducted using R (version 4.3.2). For continuous variables assumed to follow a normal distribution between the two groups, t-tests were applied. The exploration of the correlation between gene expression and immune cell components was conducted using the Spearman rank correlation test. The experimental data were plotted and analyzed using GraphPad Prism 8.0, employing the Student’s t-test for statistical significance. The threshold for statistical significance was established at a p-value <0.05.

## Result

### Identification of DEGs between MDS and normal control groups

**FIGURE 1:**
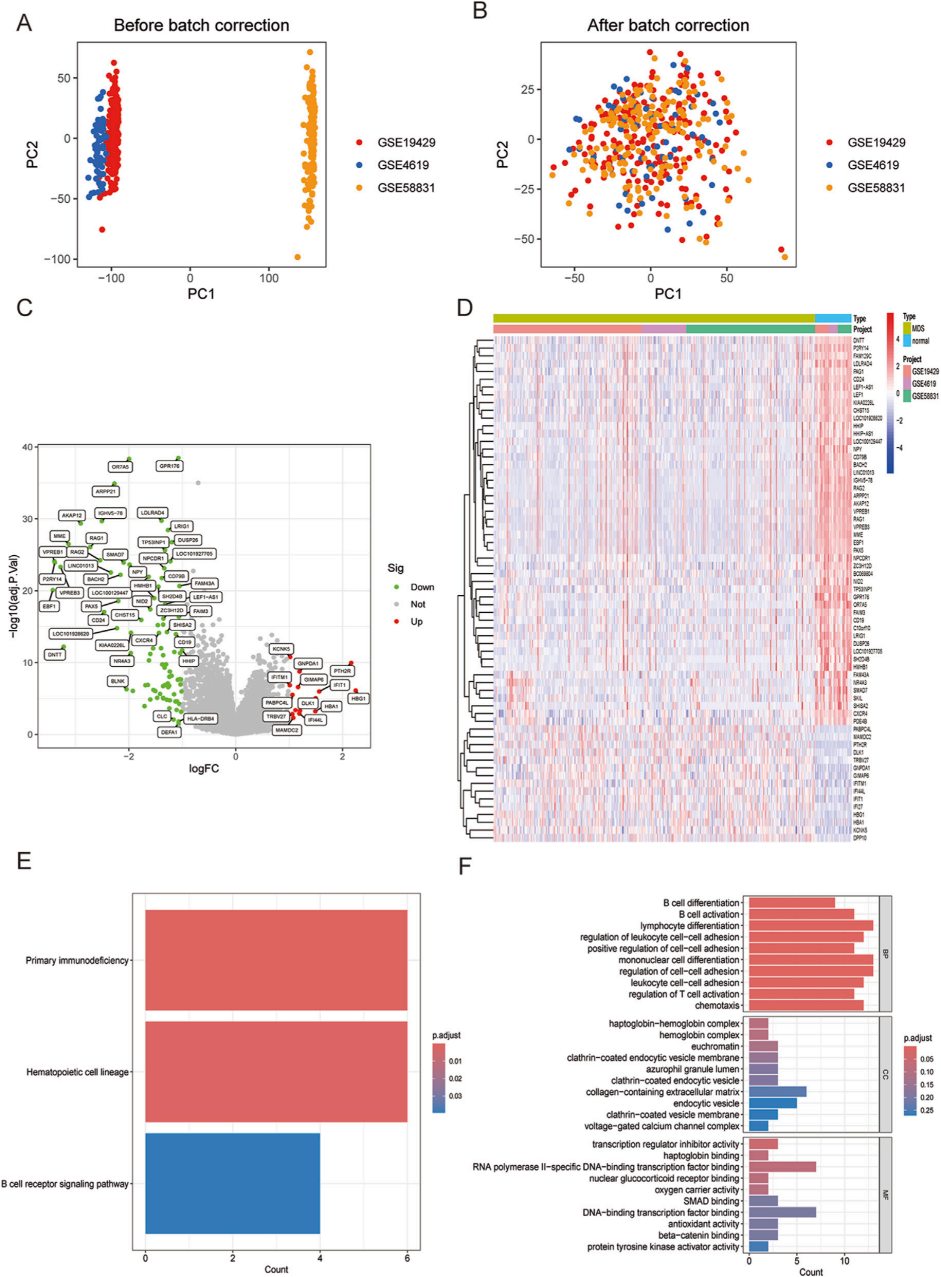
**(A)** Principal component analysis (PCA) showing the distribution of samples in each dataset. **(B)** PCA after de-batch processing showing the distribution of samples in each dataset. **(C)** Volcano plot visualizing DEGs between MDS and normal control groups. **(D)** Heatmap visualizing DEGs between MDS and normal control groups. **(E)** Enriched KEGG analysis in MDS compared with normal control groups. **(F)** Enriched GO analysis in MDS compared with normal control groups.

The datasets GSE4619, GSE19429, and GSE58831 were selected from the GEO database to constitute the training group, including 397 MDS patients and 35 normal controls. Principal component analysis (PCA) was performed on these datasets to facilitate subsequent differential gene analysis, ensuring accuracy and comparability post de-batch processing, as demonstrated in the figures below (Figures 1A,B).

Analysis of the merged and normalized datasets revealed 108 significantly differentiated genes in MDS. Volcano plots visualized the expression changes of these genes (Figure 1C), featuring 14 upregulated genes such as IFITM1, KCNK5, TRBV27, PABPC4L, MAMDC2, and 94 significantly downregulated genes including GPR176, OR7A5, ARPP21, LDLRAD4, IGHV5-78 (Figure 1D).

Subsequent KEGG analysis compared the MDS group with the normal control group, highlighting three strongly relevant signaling pathways: the primary immunodeficiency-related pathway, the hematopoietic cell regulatory signaling pathway, and the B-cell receptor signaling pathway (Figure 1E). Additionally, GO analysis identified biological processes closely associated with immune responses in terms of BP, including lymphocyte differentiation, monocyte differentiation, and regulation of cell-cell adhesion (Figure 1F). Notably, MDS represents a group of clonal diseases originating from the malignant transformation of hematopoietic stem cells (HSCs). A distinguishing feature of MDS is its ineffective hematopoiesis (Bazinet and Bravo, 2022). Furthermore, research into the bone marrow microenvironment and immune abnormalities forms a significant area in the study of MDS pathogenesis (Lee et al., 2021). The results of this study align closely with the current state of research, reinforcing the validity and credibility of our findings.

### Identification and validation of hub genes in MDS

**FIGURE 2:**
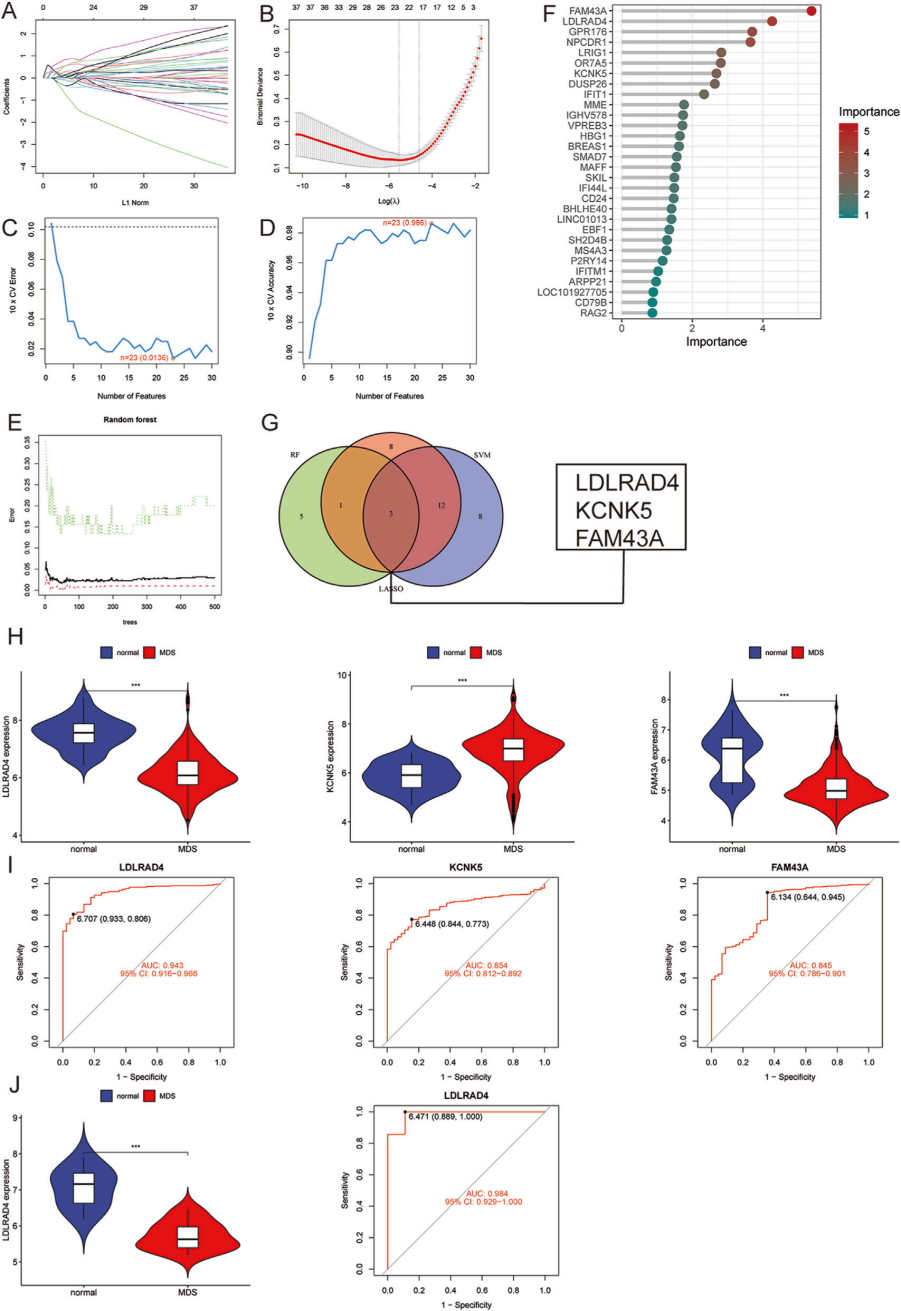
**(A)** LASSO coefficient pathway plot of hub genes related to myelodysplastic syndrome in the training group. **(B)** LASSO regression cross-validation curve. The optimal λ value was determined using 10-fold cross-validation in the training group. **(C)** Identification of 23 gene features through SVM-RFE analysis, with an error of 0.0136. **(D)** Identification of 23 gene features through SVM-RFE analysis, with an accuracy of 0.986. **(E)** Error rate confidence interval of the random forest model in the training group. **(F)** Lollipop chart showing the relative importance of genes in the random forest model in the training group. **(G)** Intersection plot of LASSO, SVM-RFE machine learning, and random forest feature genes. **(H)** Comparison of the expression levels of the three hub genes in MDS and normal control groups in the training group. **(I)** ROC analysis of the three hub genes in the training group. **(J)** Comparison of the expression level of LDLRAD4 and ROC analysis in the validation group and the normal control group in the training group.

Three machine learning methods were employed to identify hub genes closely related to MDS: LASSO regression analysis, SVM-RFE, and the random forest model. These methods were utilized to screen key candidates among differentially expressed genes. Initially, LASSO regression analysis was used to screen for differentially expressed genes, and the stability and reliability of the results were ensured through a 10-fold cross-validation method. Ultimately, 23 key genes were identified (Figures 2A,B). Subsequently, the SVM-RFE method was employed to identify 23 key genes, with an error rate of 0.0136 and an accuracy rate of 0.986 (Figures 2C,D). Ultimately, a random forest model was used to rank the importance of all genes, focusing on those with importance scores greater than 1 (Figures 2E,F). To further enhance the reliability of the results, an intersection analysis was performed on the key genes identified by each method, ultimately pinpointing three pivotal genes closely associated with MDS: LDLRAD4, KCNK5, and FAM43A (Figure 2G).

An in-depth analysis was conducted to validate the reliability of the three hub genes, focusing on their expression levels in the training group. It was found that the expression levels of LDLRAD4 and FAM43A were significantly downregulated in the MDS group compared to the normal control group, while KCNK5 showed a significant uptrend (Figure 2H). Subsequently, the area under the receiver operating characteristic curve (AUC-ROC) was calculated for these three genes. The AUC values were: 0.943 for LDLRAD4 (95% CI: 0.915-0.966), 0.854 for KCNK5 (95% CI: 0.812-0.892), and 0.845 for FAM43A (95% CI: 0.786-0.901) (Figure 2I). Given that the AUC values of these genes were all above 0.8, this indicates their high diagnostic efficiency in predicting MDS.

Based on the aforementioned analyses, LDLRAD4 was selected as the core hub gene for this study. To verify the stability and reliability of LDLRAD4, the GSE2779 dataset from the GEO database was employed as the validation set. The validation set included 17 normal controls and 159 MDS cases. Re-analysis of LDLRAD4’s expression levels and AUC-ROC values in the validation set revealed high consistency with the training set results. Specifically, LDLRAD4’s expression levels were significantly downregulated in MDS patients, with an AUC value of 0.984 (95% CI: 0.929-1.000) (Figure 2J). This result not only confirms LDLRAD4’s reliability as a hub gene but also suggests its potential applications in the diagnosis and prediction of MDS.

### Hub gene GSVA and co-expression analysis (LDLRAD4)

**FIGURE 3:**
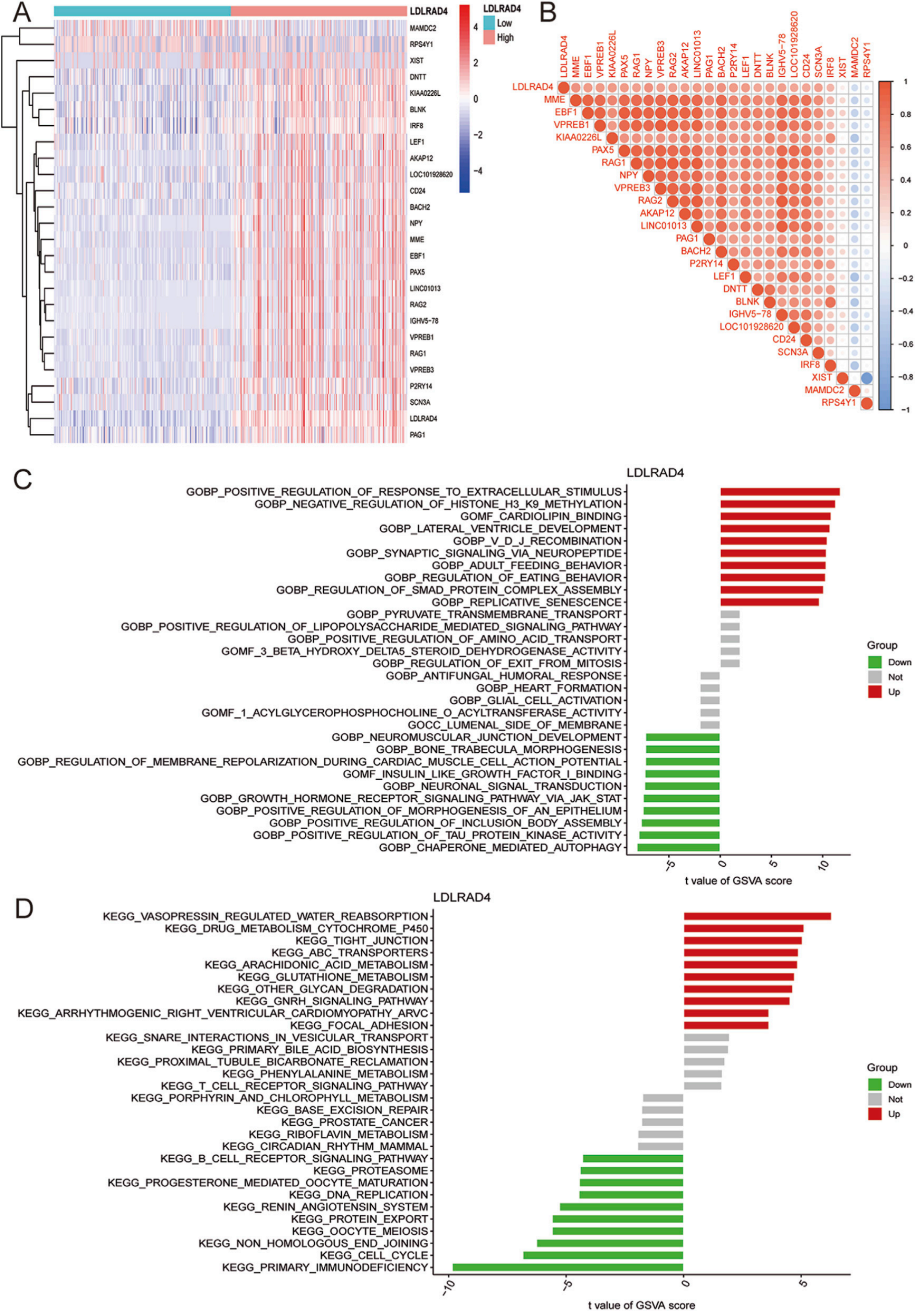
**(A)** Heatmap showing differential genes after grouping LDLRAD4 by high and low expression. **(B)** Correlation analysis between LDLRAD4 and differential genes: red (positive correlation), blue (negative correlation). **(C)** GSVA of GO items between high and low expression groups of LDLRAD4 in MDS. **(D)** GSVA of KEGG items between high and low expression groups of LDLRAD4 in MDS.

Having identified LDLRAD4 as the focal point of this study, its function and mechanism were explored in depth. LDLRAD4’s mRNA expression level in MDS was categorized based on the median value, dividing it into low and high expression groups. Subsequent differential analysis of these groups led to the identification of 26 differential genes, including MAMDC2, RPS4Y1, MME1, and EBF1 (Figure 3A). Co-expression analysis was conducted on the obtained differential bases, sorting them by correlation strength. Results indicated a positive correlation of LDLRAD4 with genes including MME, EBF1, VPREB1, KIAA0226L, and PAX5, and a negative correlation with MAMDC2 and RPS4 (Figure 3B).

To further elucidate LDLRAD4’s role in MDS, GSVA analysis of GO and KEGG pathways was performed for its high and low expression groups. In terms of GO, the high-expression group showed enrichment in: GOBP: Positive Regulation of Response to Extracellular Stimulus, GOBP: Negative Regulation of Histone H3 K9 Methylation, and GOMF: Cardiolipin Binding, among others. The low-expression group showed enrichment in: GOBP: Neuromuscular Junction Development, GOBP: Bone Trabecula Morphogenesis, and GOBP: Regulation of Membrane Repolarization During Cardiac Muscle Cell Action Potential, among others (Figure 3C). KEGG enrichment analysis revealed for the high-expression group: Vasopressin Regulated Water Reabsorption, Focal Adhesion, Arrhythmogenic Right Ventricular Cardiomyopathy (ARVC), GnRH Signaling Pathway, Other Glycan Degradation, and others; for the low-expression group: Primary Immunodeficiency, Cell Cycle, Non-Homologous End Joining, and others (Figure 3D).

Given the GO analysis results, an in-depth exploration of the regulation of Smad protein complex assembly, a critical biological process, is warranted. Additionally, enrichment in the primary immunodeficiency-related pathway and B-cell receptor signaling pathway in KEGG analysis aligns with previous analyses of MDS samples. These pathways are particularly intriguing. A substantial proportion of genes correlating with LDLRAD4 have yielded significant research outcomes in immunity. This suggests the promise of focusing on immunity in this direction for future research endeavors.

### GSEA analysis of hub genes

**FIGURE 4:**
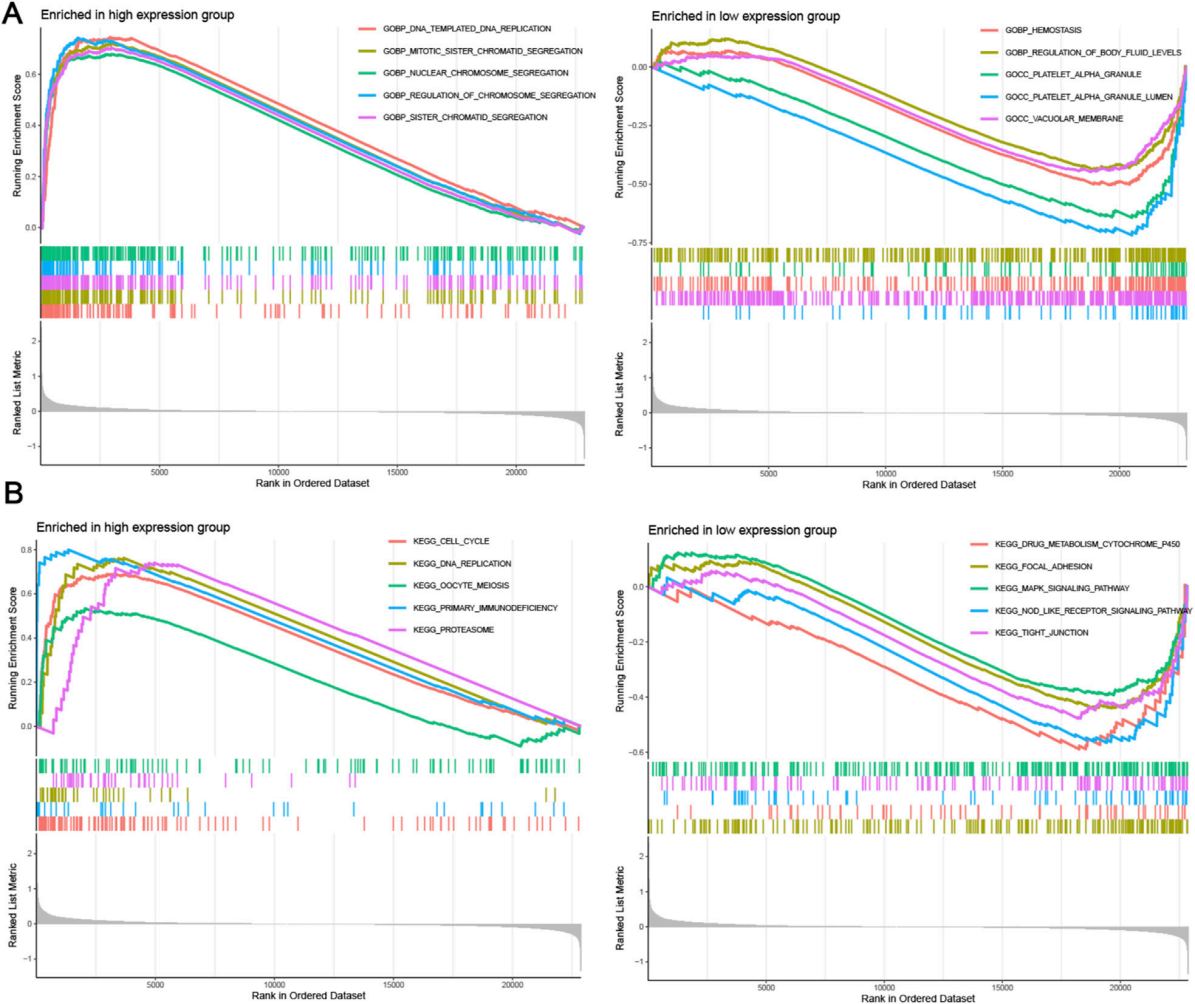
**(A)** GESA analysis of GO items for high and low expression groups of LDLRAD4 in MDS. **(B)** GSEA analysis of KEGG items for high and low expression groups of LDLRAD4 in MDS.

To conduct a more comprehensive analysis of LDLRAD4’s function and mechanism, GSEA was again employed to analyze the high and low expression groups of LDLRAD4 in terms of GO and KEGG. Regarding GO, the high-expression group was enriched in GOBP DNA Templated and DNA Replication, GOBP Mitotic Sister Chromatid Segregation, and others; the low-expression group showed enrichment in GOBP Hemostasis, GOBP Regulation of Body Fluid Levels, and others (Figure 4A). In KEGG analysis, the high-expression group was enriched in Cell Cycle, DNA Replication, Oocyte Meiosis, and others; while the low-expression group showed enrichment in Drug Metabolism Cytochrome P450, Focal Adhesion, MAPK Signaling Pathway, and others (Figure 4B).

Many GO analysis results, including DNA replication, chromosome segregation, and mitosis, were found to be associated with cell proliferation processes. Results from KEGG analysis suggest that LDLRAD4 may influence cell cycle-related pathways, corroborated by the GO analysis. Thus, it is hypothesized that LDLRAD4 could regulate the cell cycle through its impact on DNA templating and replication processes. Additionally, pathways aligning with the GEVA analysis results were identified, including those related to primary immunodeficiency disease and cell cycle. This finding further underscores the significance of LDLRAD4 within these pathways.

### Analysis of immune infiltration in MDS and correlation between hub genes and infiltrating immune cells

**FIGURE 5:**
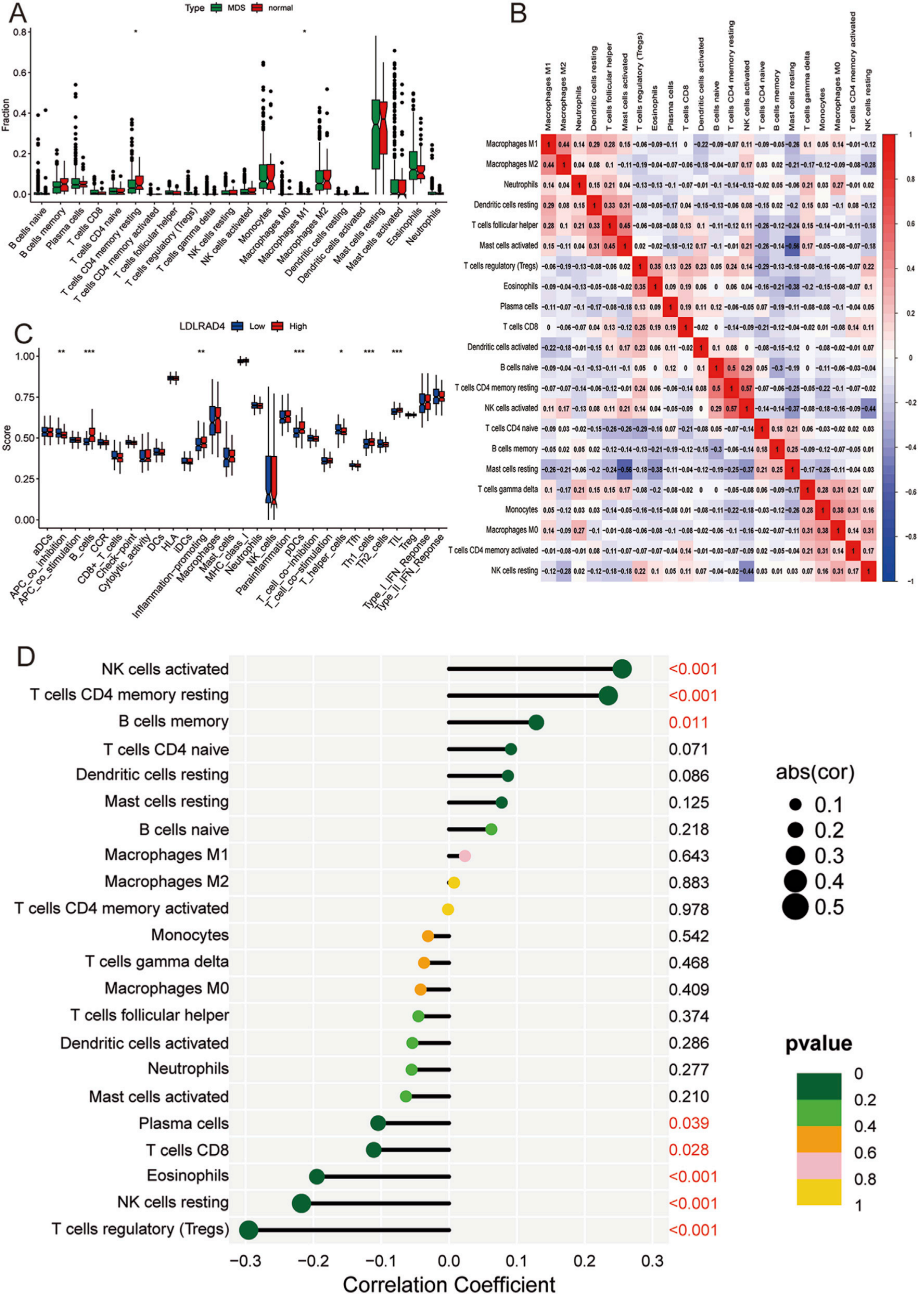
**(A)** Box plot of immune cell infiltration between MDS and normal control groups. **(B)** Correlation analysis between different immune cells: red (positive correlation), blue (negative correlation). **(C)** Box plot showing the difference in immune cell-related functions between high and low expression groups of LDLRAD4. **(D)** Lollipop chart showing the correlation between LDLRAD4 and different immune cells.

Given the close association of MDS with immunity in terms of LDLRAD4’s function and pathway enrichment, the CIBERSORT algorithm was employed to investigate the infiltration abundance of 22 immune cell types in the training set samples. Comparisons between the MDS and normal groups revealed significant differences in the abundance of T cells CD4 memory resting and Macrophages M1 (P < 0.05) (Figure 5A). To elucidate the complex interactions among immune cells, correlations between various immune cell types were investigated. Findings indicated a strong positive correlation between M1 and M2 macrophages, and a significant negative correlation with resting dendritic cells. Additionally, the strongest positive correlation was found between T cells CD4 memory resting and activated NK cells, and the most significant negative correlation with resting mast cells (Figure 5B).

Furthermore, ssGSEA was utilized to analyze the degree of immune cell activation and function in the high and low expression groups of LDLRAD4. Results revealed significant elevations in B cells, pDCs, Th1 helper cells, TIL, and inflammation-promoting in the high-expression group of the MDS group. Conversely, APC_co_inhibition and T helper cells showed significantly higher expression in the low-expression group (Figure 5C).

The relationship between infiltrating immune cells and LDLRAD4 was evaluated, revealing positive correlations with activated NK cells, T cells CD4 memory resting, and memory B cells, and negative correlations with Plasma cells, CD8 T cells, Eosinophils, resting NK cells, and Tregs (Figure 5D). These findings complement the conclusions from GO, KEGG, and co-expression analyses of LDLRAD4, enhancing the understanding of its association with immunity. Additionally, they offer crucial insights into the mechanisms of immune dysfunction and disease progression in MDS patients.

### Overexpression of LDLRAD4 inhibits the proliferation of SKM-1 cells and promotes apoptosis

**FIGURE 6:**
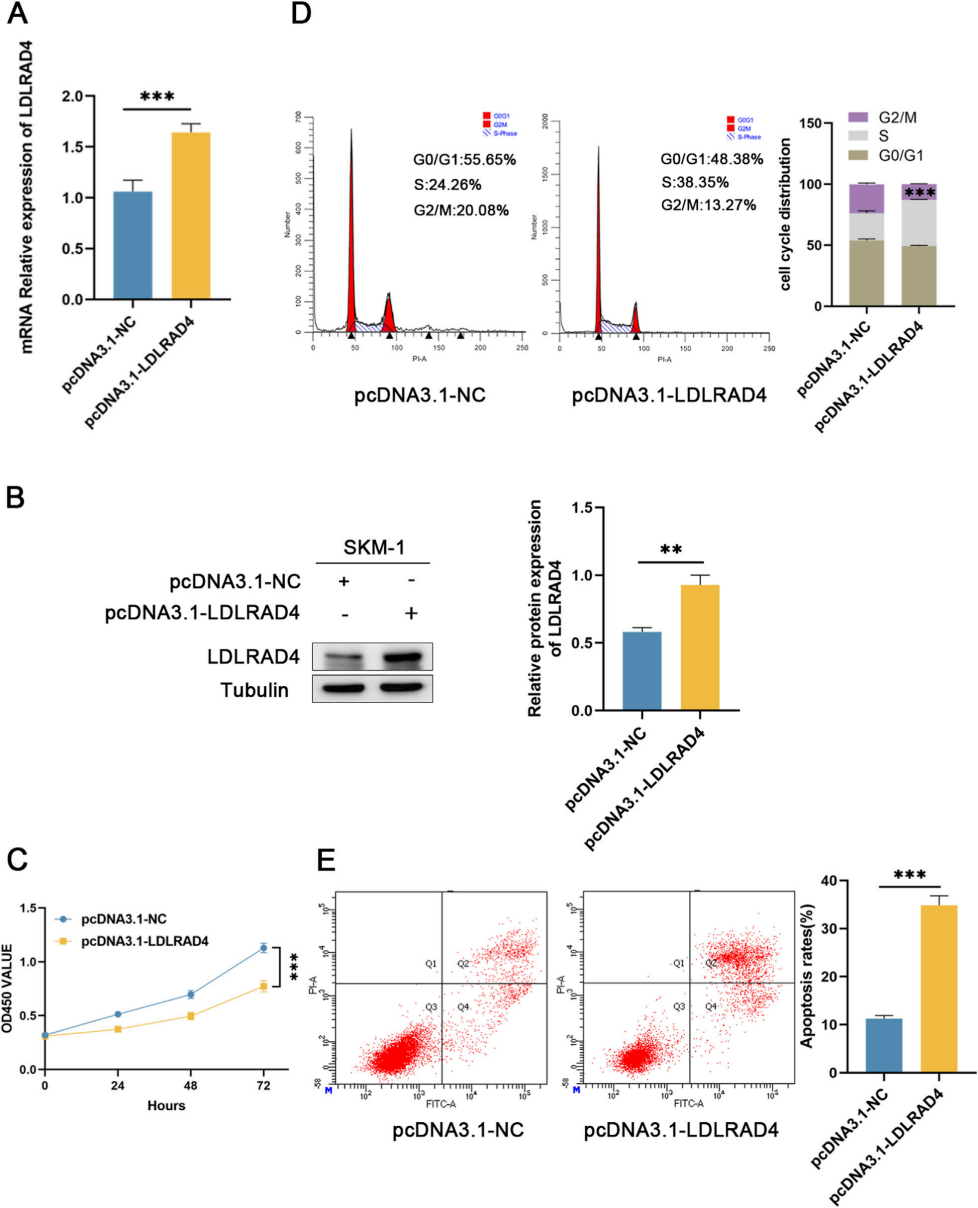
**(A)** qRT-PCR was used to detect the mRNA level of pcDNA3.1-LDLRAD4 compared to the control. **(B)** Western blotting was employed to detect LDLRAD4 protein levels following transfection with pcDNA3.1-LDLRAD4, compared with the control. **(C)** The CCK-8 assay showed that LDLRAD4 overexpression decreased SKM-1 proliferation compared to the control. **(D)** LDLRAD4 overexpression resulted in cell cycle arrest at the S phase. **(E)** Overexpression of LDLRAD4 results in an increased rate of apoptosis in SKM-1 cells.

The analysis indicates that LDLRAD4 is significantly downregulated in MDS patients. To further investigate the impact of LDLRAD4 on the biological functions of MDS cell lines, we selected SKM-1 as our *in vitro* cellular model. We established a control group (pcDNA3.1-NC) and an overexpression group (pcDNA3.1-LDLRAD4). Western blotting and qRT-PCR revealed that the LDLRAD4 levels in the overexpression group were significantly higher compared to the control group (Figures 6A,B). Initially, we employed the CCK8 assay to evaluate the impact of LDLRAD4 on the viability of SKM-1 cells. The results indicated that overexpression of LDLRAD4 in SKM-1 cells significantly inhibited their proliferative capacity compared to the control group (Figure 6C). Subsequently, using flow cytometry, we found that compared to the control group, the overexpression group had cells arrested in the S phase, with a reduction in G2/M phase cells, indicating inhibited cell proliferation (Figure 6D). Finally, flow cytometry was used to assess apoptosis, and the overexpression group showed a significantly higher rate of cell apoptosis compared to the control group (Figure 6E). These findings suggest that LDLRAD4 may be a gene that inhibits the proliferation of MDS cells and promotes apoptosis.

## Discussion

Myelodysplastic syndromes (MDS) are often referred to as a ‘gray disease’ in the field of blood diseases due to their low public awareness and unclear pathogenesis. The insidious onset and lack of specific symptoms in the early stages can cause many patients to miss the best treatment opportunities. MDS also has the potential to transform into acute myeloid leukemia (AML), which can have serious health consequences (Bazinet and Bravo, 2022). Early identification and intervention for MDS are crucial for improving patients’ quality of life. Elucidating its pathogenesis is also urgent. In recent years, the connection between clinical and bioinformatics has become increasingly close, enabling us to more accurately identify biologically meaningful biomarkers.

Comprehensive bioinformatics and machine learning techniques were utilized to identify hub genes, resulting in the identification of 108 differentially expressed genes (DEGs) with significant differences. Functional and pathway enrichment analyses demonstrated a robust association with the immune response. Exploration of the DEGs through LASSO regression analysis, SVM-RFE, and the random forest model led to the identification of three pivotal genes: LDLRAD4, KCNK5, and FAM43A. ROC curve analysis determined the diagnostic value of these genes, confirming their significance.

Immunotherapy has recently emerged as a promising approach in the treatment of MDS. Our analysis integrated autoimmune abnormalities in MDS patients with the enrichment of primary immunodeficiency-associated pathways, utilizing DEGs to examine immune infiltration in the MDS group relative to the normal group. Significant differences were observed in a broad spectrum of immune cells between the two groups. The focus was placed on the vital role of macrophages within the bone marrow microenvironment, among other immune cells. Studies have indicated that MDS induces abnormal alterations in the bone marrow microenvironment, resulting in varied macrophage polarization (Murray and Wynn, 2011). This analysis aligns with current research, underscoring the need for further exploration into macrophages’ role and mechanisms in MDS.

The KCNK5 gene encodes TASK-2, a critical potassium channel involved in renal function, and has potential roles in preventing ischemic neurodegeneration and in breast cancer treatment (Warth et al., 2004; Heitzmann et al., 2008; Alvarez-Baron et al., 2011; Gob et al., 2015). Recent investigations into this gene have contributed significantly to immunological research. Notably, the upregulation of KCNK5 is linked to T cell proliferation (Kirkegaard et al., 2016), and bidirectional potassium channels, potentially involving KCNK5, may be integral in NK cell activation and effector functions (Schulte-Mecklenbeck et al., 2015). Yet, this area remains largely unexplored in blood disorder studies. Our study is unique in analyzing the significant upregulation of KCNK5 in MDS, suggesting it as a potential emerging risk factor. In conclusion, TASK-2, encoded by KCNK5, plays a significant role across various biological domains. This discovery opens new avenues for the treatment and diagnosis of related diseases.

Currently, limited research has been conducted on FAM43A, which is primarily based on bioinformatics analysis. FAM43A is hypothesized to potentially serve as a new prognostic biomarker and therapeutic target for diseases such as sepsis or triple-negative breast cancer (Shen et al., 2024; Chen et al., 2011). In this study, the FAM43A gene was identified through various machine learning analyses. The study found that the expression level of FAM43A was significantly downregulated in MDS patients. This finding suggests a new research direction for future studies on targeted drugs for the treatment of MDS.

This paper focuses on LDLRAD4, which has been shown to possess superior diagnostic value compared to KCNK5 and FAM43A, as evidenced by the ROC curve. Prior studies have associated this gene with psychiatric disorders (Kikuchi et al., 2003b; Kikuchi et al., 2003a),and indicate that it shares a similar function with TMEPAI as a negative regulator of TGF-β signaling (Nakano et al., 2014). In the realm of tumor research, LDLRAD4 presents unique research potential. Zhenxing Liu et al. reported that LDLRAD4 interaction with Nedd4, an E3 ubiquitin ligase, promotes the proliferation and migration of hepatocellular carcinoma cells (Liu et al., 2017); Yuko Ito et al. showed that non-genotoxic hepatocellular carcinogens cause the downregulation of oncogenic LDLRAD4 in rat livers, which leads to the disruptive activation of TGF-β signaling (Ito et al., 2020). Additionally, it has been suggested that the mRNA expression level of LDLRAD4 could be an independent prognostic factor for GIST, a hypothesis requiring further confirmation (Xie et al., 2020). In summary, LDLRAD4 exhibits a novel role in tumorigenesis and could potentially be targeted for the treatment of hepatocellular carcinoma. However, a direct association with blood disorders remains unestablished.

This study entailed conducting GO and KEGG analyses on the LDLRAD4 high and low expression groups using GSVA. The GO analysis results indicated a close association between LDLRAD4 and the regulation of Smad protein complex assembly. Prior research has demonstrated that LDLRAD4 has a negative regulatory effect on the TGF-β signaling pathway (Nakano et al., 2014). The TGF-β signaling pathway entails the activation and phosphorylation of the TGF-β receptor, initiating a regulatory cycle of activating and inhibiting Smad proteins. This regulatory loop amplifies the response of the TGF-β signaling pathway through a negative feedback mechanism or constitutive activation. This process is directly associated with myelosuppression and null erythropoiesis in MDS (Blank and Karlsson, 2015; Larsson and Karlsson, 2005; Blank and Karlsson, 2011). Based on these findings, it is hypothesized that LDLRAD4 could be involved in regulating the pathogenesis of MDS by influencing the TGF-β signaling pathway. This hypothesis further posits LDLRAD4 as a potential therapeutic target, warranting exploration in subsequent studies. Additionally, it was discovered that the genes EBF1 and PAX5, correlated with LDLRAD4, play crucial roles in B cell development and function (Yang et al., 2016; Inagaki et al., 2016). This finding offers a theoretical basis for predicting the potential impact of LDLRAD4 on MDS in terms of immunity. This can also guide subsequent studies. Interestingly, upon re-analyzing the GO and KEGG aspects of LDLRAD4 using GSEA, similar results were obtained. Furthermore, the study identified the MAPK pathway, a classical signaling pathway responsible for myeloid proliferation under physiological conditions and aberrantly activated in myeloproliferative diseases (Rocca et al., 2018). Mutations in NRAS, JAK2, and CSF3R, key signaling components, have been shown to aberrantly activate the MAPK pathway. Consequently, inhibitors targeting these signaling components have found clinical application. These inhibitors demonstrate potential, particularly in treating patients with atypical chronic granulomatous leukemia (aCML), a rare MDS/myeloproliferative neoplasm (MPN). Based on this investigation, LDLRAD4 emerges as a promising target for treating MDS. Its potential applications extend beyond merely treating aCML (Rocca et al., 2018).

Extensive biochemical analysis of LDLRAD4 has unveiled its research value in immunity. Consequently, we analyzed its immune function and correlation with immune cells. According to the results of correlation analysis among different immune cells, LDLRAD4 has the strongest correlation with NK cells. NK cells constitute a key component of the immune system and function as the first line of defense for the organism. They have multiple functions, including anti-tumor and anti-virus activities, regulation of immune balance, participation in tissue repair, and the ability to rapidly kill virus-infected and tumor cells, even in the absence of prior immune activation (Sivori et al., 2021). Research has shown that in patients with MDS, NK cells in the bone marrow exhibit a decreased killing capacity (Fischer et al., 2007; Schonberg et al., 2011). Additionally, there exists a reduced number of NK cells, an imbalance of subtypes, and a decrease in activating receptors. These factors might contribute to the inability of NK cells to effectively clear MDS malignant clonal cells, thus promoting disease progression. In contrast, our observations revealed that LDLRAD4 was significantly under-expressed and strongly positively correlated with NK cells in MDS patients. Consequently, we hypothesized that LDLRAD4 might influence the course of MDS disease by regulating the function of NK cells. We also explored the possibility that MDS might alter the number and efficacy of NK cells by regulating the expression of LDLRAD4. These uncharted territories offer new perspectives for future research and inspire the potential to develop immunotherapeutic strategies based on NK cells. This study aims to probe the interactions between LDLRAD4 and immune cell subsets, and their potential roles in immunomodulation in the context of MDS. Future studies ought to delve into the mechanisms of these interactions and their synergistic effects on the pathologic process of MDS. This could provide new strategies and approaches for the clinical treatment of MDS. Subsequently, utilizing the SKM-1 cell line as an *in vitro* model for MDS, we found that the overexpression of LDLRAD4 led to inhibited proliferation, cell cycle arrest at the S phase, and increased apoptosis in SKM-1 cells. The experimental findings suggest that LDLRAD4 has an inhibitory effect on the proliferative activity of MDS cells; however, the precise molecular mechanisms underlying this regulation require further investigation.

In summary, this study analyzed the key genes associated with MDS, namely, LDLRAD4, FAM43A, and KCNK5, using bioinformatics and machine learning methods. The study focused on unveiling the central role of LDLRAD4 in the pathological process of MDS, with special attention to its regulatory role in the TGF-β signaling pathway and its association with B-cell development and the MAPK signaling pathway. The study offers new targets and therapeutic strategies for the treatment of MDS. Additionally, the study highlights the significant role of NK cells in MDS. It suggests that LDLRAD4 may regulate the disease process by affecting NK cell function, thus offering a new avenue for immunomodulation-based therapeutic strategies. Although this study provides a comprehensive bioinformatic analysis of LDLRAD4, covering its functional, pathway, and immunological roles, it is important to acknowledge its limitations. For instance, the feature selection techniques we employed, such as LASSO, SVM-RFE, and RF, can be sensitive to the specific composition of the dataset. Furthermore, the pronounced heterogeneity of MDS, with its numerous subtypes characterized by distinct classifications, blast counts, and genetic profiles, presents a challenge for a unified analysis. Our current dataset does not permit a detailed investigation into each subtype. Crucially, our findings still require validation with clinical samples and further exploration of the specific mechanistic role of LDLRAD4 in MDS using *in vitro* models. Future investigations will address these limitations to enable a deeper understanding.

## Data Availability

The datasets generated during and/or analysed during the current study are available from the corresponding authors on reasonable request.
